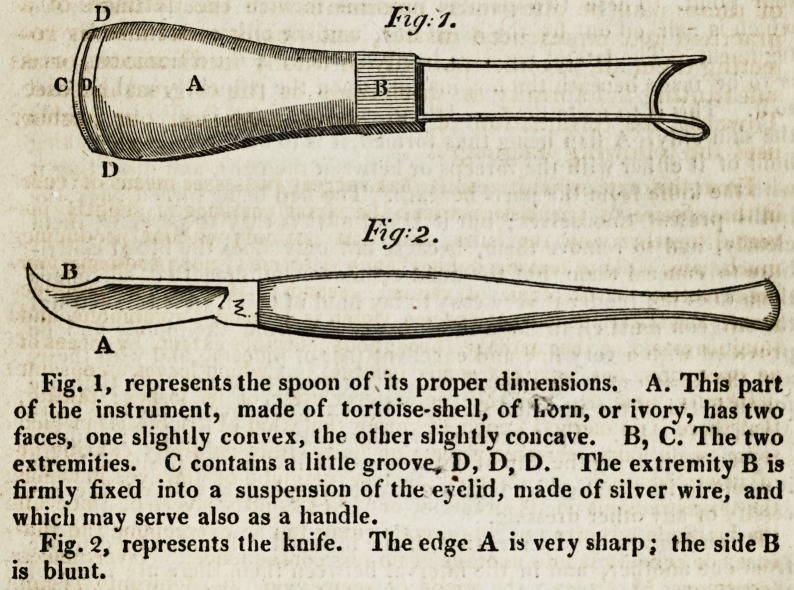# Book Reviews

**Published:** 1826-04

**Authors:** 


### CRITICAL ANALYSIS
### OF
### ENGLISH AND FOREIGN LITERATURE,
### RELATIVE TO THE VARIOUS BRANCHES OF
### Medical Science.

Quæ laudanda forent, et quæ culpanda, vicissim

Illa, prius, cretâ; mox hæc, carbone, *notamus.*—PERSIUS.

### DIVISION I.

### ENGLISH.

ART. I.—*An Introduction to the Use of the Stethoscope; with its Application to the Diagnosis in Diseases of the Thoracic Viscera: including the Pathology of these various Affections.* By WILLIAM STOKES, M.D.—12mo. pp. xiii. 226. Edinburgh: Maclachlan and Stewart, 1825.

WHEN LAENNEC'S valuable work appeared some years ago, the readers of this Journal were made acquainted with it in various elaborate reviews. We at that time obtained a stethoscope, (one of the first which was brought to this country,) and endeavoured to judge for ourselves of its merits in assisting diagnosis. The result of our trials was not favourable; but we were cautious in forming an opinion, as we conceived that our not distinguishing with precision all the sounds described by Laennec, might have arisen from some personal defect in the sense of hearing. During the five or six years which have intervened, several works have been written on the subject, both abroad and in this country; the last, and one of the best, of which we have selected, for the purpose of again calling the attention of our readers to the subject. This we do, not so much from having altered our own opinions, as from regarding it to be our duty to make those who peruse our Journal acquainted with the best works of the day, and put them in a situation to judge for themselves of the validity of the doctrines they contain. That the author before us entertains very different opinions of the utility of the stethoscope, it is almost unnecessary to say; for the work is even dedicated to a gentleman, on account of his having paid "unremitting attention to the light which mediate auscultation is now throwing on the obscurity of the disease;" and, in his Preface, he continues—

"I might here enter into a long dissertation on the utility of the stethoscope, but such is not my intention. It is a common objection to the use of this instrument, that it leads to no practical results, and therefore that it is more useful to the pathologist than to the physician. But those who make use of such an objection, only betray their ignorance of the use of the stethoscope, and, like unjust judges, pronounce sentence without examining into the merits of the case. The stethoscope, besides its vast importance in the diagnosis of a most difficult class of diseases, does lead to many useful practical results. Let us take the cases of pneumonia and of pleurisy, two of the most common and severe affections of the thoracic cavity, where a daily examination by means of the stethoscope points out the progress of the disease, its exact seat, the effect of our remedies, the necessity of their repetition, or the utility of their omission. In circumscribed pleurisy, in wounds of the thorax, its utility is undeniable. From ignorance of its application, displacement of the heart, arising from a pleuritic effusion, has been mistaken for dilatation of that organ, while the original disease was entirely overlooked. Pleurisy has been mistaken for rheumatism, and a critical diaphoresis has been checked in pneumonia. In confirmed phthisis, when the hopes of the sufferer's friends are excited by an ignorant practitioner, the physician, with the aid of the stethoscope, has at least the melancholy advantage of saving to those friends the pangs of disappointed hope, and to the patient himself the torture of useless remedies. By means of the stethoscope we can detect latent inflammatory affections of the pulmonary organs, long before they have become evident from their external symptoms. These are cases where a practitioner ignorant of mediate auscultation would be completely at a loss. I could adduce a host of other instances, but refrain from doing so, in the firm conviction that such will not be required by any one who has used the stethoscope in ten cases of thoracic disease. Even without reference to actual disease, is it not a great practical advantage that, in doubtful cases, we can explore the hidden recesses of the thorax, and say with confidence to our patient, There is no disease here?" (P. x.—xii.)

In this paragraph, the first illustration of the practical utility of the stethoscope consists in its application to pleurisy and pneumonia, in which diseases, we are told, it points out the exact seat of the inflammation—its progress—the effect of the remedies, and the necessity of repeating or omitting them. Now, without denying that the instrument may occasionally be useful in the diagnosis of obscure cases, we must protest against the absurdity of attributing to it powers which can only result from practical experience, grounded on general therapeutic principles. In cases of pneumonia and pleurisy, every practitioner is, or ought to be, able to judge sufficiently of the seat of the disease, from its symptoms; while he who trusts to any other guide, in the administration of his remedies, than the functional and constitutional derangement of his patient, may possibly be a good pathologist, but will certainly make a very sorry practitioner. Its utility in wounds of the thorax is said to be "undeniable;" we shall not, therefore, venture to dispute it: but, with regard to the next illustration, that displacement of the heart from effusion may be mistaken for dilatation of that organ, it is obviously a possible case only, and not a probable one. We can only say, that the man who made such a mistake without the stethoscope, would be very likely to fall into the same error with it. "Pleurisy has been mistaken for rheumatism." Very possibly; and, where the symptoms are such as to render the application of the stethoscope necessary to distinguish them, we advise our readers still to treat the case as one of pleuritic inflammation—say the stethoscope what it may. Lastly, we come to "confirmed phthisis;" and here the recommendation of the instrument is more equivocal than in any of the preceding cases. It is supposed that "an ignorant practitioner" misleads the friends, and tortures the patient with "useless remedies." Under these circumstances, "the physician, with the aid of the stethoscope," detects the disease, and consequently the inutility of "useless remedies." Now, Dr. Stokes surely must admit, that a physician, who is not "ignorant," would make both of these notable discoveries quite easily without the stethoscope. That man must be ignorant indeed who requires any such assistance to detect a case of "confirmed phthisis." Yet we object not to this manner of stating the question, for we believe that it is in general the true one. We mean that where any decided superiority is shown by the "physician with the aid of the stethoscope," it is when (as in the case here supposed) he is placed in opposition to "an ignorant practitioner."

We are next told that, by means of this instrument, we can detect latent inflammatory affections, before they become manifest to our ordinary means of scrutiny: this, if true, is a decided advantage. We do not positively deny the position, but we doubt it: at present it stands as an assertion, without sufficient proof. Not long ago, a physician in this metropolis, who patronises the stethoscope, mentioned a case of hydrothorax, in which he had discovered the effusion by means of this instrument. He was asked, Had the patient any difficulty of breathing? Yes.—Could she lie flat in bed? No.—Any purpleness of the lips? Yes.—Any swelling of the ankles? Yes.—Any scantiness of the urine? Yes.—Then, rejoined the querist, the only difference between us is, that I should have formed the same conclusion without the stethoscope which you did with it.

But Laennec is always referred to, and his extraordinary skill in thoracic complaints triumphantly quoted, as setting all arguments at rest. But this illustration makes nothing in favour of the stethoscope: in our opinion, indeed, it is against it; for it is to be remembered that Laennec was a great pathologist before this instrument was heard off, and, instead of the stethoscope giving to him the knowledge he now possesses, he gave to it the reputation derived from thirty years' assiduous and successful cultivation of pathology, and all the diagnostic acumen necessarily resulting therefrom.

We are quite aware that our scepticism will be laid, by the friends of mediate auscultation, to the charge of our ignorance. Be it so: but at the same time we must claim the privilege, in our turn, just to hint at the Tale of the Tub; and we must add, ("not to speak it profanely,") that our northern neighbours, who are marvellously smitten with this discovery, have always been rather partial to tubs. It is justly observed by Dr. Stokes, that, "in the history of mankind, it will be found that no great discovery, or probable conjecture, was ever promulgated, without encountering the most bitter opposition." Our opposition, however, is not bitter; but merely the legitimate misgivings of men who have tried the instrument, who have seen others try it, who have read what has been written upon the subject, who have conversed with those whom they regarded as better qualified to judge than themselves,—the result of all which has been a doubt whether a practitioner of the ordinary range of education, talent, and experience, cannot detect diseases of the chest as well by the means heretofore adopted, as one who places his confidence in mediate auscultation. We shall, however, give to our readers the benefit of the doubt, and lay before them the most important parts of Dr. Stokes's work, which, we repeat, is clear, concise, and unassuming.

*Percussion* occupies the first portion of the volume.

"The sound heard upon striking the chest is always proportional to the size of this cavity, and the thickness and elasticity of its parietes. It varies according as we strike on a point covered with soft and thick parts; according to the state of emaciation or infiltration of the cellular substance, the posture of the patient, the part struck, and the manner of practising the percussion.

"A clear sound, which has been compared to that produced by striking an empty barrel, is obtained anteriorly when we strike on the clavicles, on the space two or three inches below them, on the entire surface of the sternum, and the neighbouring parts of the costal cartilages.

"Laterally, the axilla, and the space for three inches below it, are the places where the clearest sound is obtained: on the right side, from the fourth rib, and sometimes even the third, to the inferior part of the chest, the sound becomes less clear on account of the vicinity of the liver; while, on the left, it is often clearer from the proximity of the stomach, especially when this viscus is distended with air.

"Posteriorly, by striking on the line of the costal angles, and, in thin subjects, on the spaces superior or inferior to the spine of the scapula, and also on the spine of the scapula itself, a clear and distinct sound is obtained; but we learn nothing from using percussion on the thick muscular bed which fills the vertebral grooves.

"It is almost unnecessary to mention that, *cæteris paribus*, the chest of an emaciated person will be more sonorous than that of an individual loaded with fat, or whose muscles are large and soft. In a patient labouring under infiltration of the cellular substance of the thoracic parietes, no conclusion can be drawn from percussion. It is necessary, in order to hear and judge of the sound from percussion, that the patient shall be placed sitting in his bed, and that the part which we wish to examine shall be stripped of all clothing. In examining the anterior part of the chest, the arms are to be held backwards; when it is the posterior, they are to be crossed upon the chest, and the patient is to bend forwards. The object of these different positions is to stretch the muscles which cover the parietes of the thorax.

"In order that percussion, apparently so simple an operation, shall lead to truly useful results, a great number of precautions are necessary. In the first place, the fingers are to be held in a state of demiflexion; their extremities should be in the same line; the operator is to strike with an equal and moderate force on similar parts, and in the same manner,—that is to say, letting the extremities of the fingers fall perpendicularly on the part under examination.

"A too strong percussion excites pain, an unequal one gives results unworthy of confidence: this will also happen if we strike on dissimilar parts,—as, for instance, alternately a rib and an intercostal space; or if the fingers are held in different positions at opposite sides. It is also necessary not to examine at once all the points on one side, before passing to the examination of the opposite one, as we are thus liable to forget the particular results which we may have obtained; it is better to examine in turn the corresponding parts of each side.

"The alterations of sound which take place in disease, may be reckoned as three in number,—viz. dull, obscure, or clearer than natural; in some cases the sound is wanting altogether. Whenever the lung loses its elasticity, and becomes engorged, without however entirely losing its permeability, the sound will become dull or obscure, according as the sanguineous infiltration of the pulmonary tissue is more or less considerable. This alteration is produced by the first degree of pneumonia, and by œdema of the lung. The sound disappears altogether in two cases; first, when the lung loses its permeability, from the abundant exhalation of blood into the areolæ and the interlobular cellular tissue, thus becoming dense, and resembling much the appearance of a portion of liver; and, secondly, when it is compressed and pushed inwards, either by some growth accidentally developed in its own substance, or in the cavity of the pleura; or when the latter is filled by any liquid.

"In these cases, a greater or less portion of the side affected still retains its sound on percussion, according as the hepatisation, accidental tumor, or effusion, may be more or less considerable.

"The sound is louder than in the state of health, when (if I may be allowed the expression) the pulmonary tissue is rarefied, as in emphysema, or when the cavity of the pleura is filled by air or other gaseous fluids." (P. 3—7.)

It is acknowledged, however, by M. Laennec, and probably not denied by any of his followers, that percussion affords but a very imperfect index to the state of the thoracic viscera. It gives no mark by which pneumonia, pleurisy, or hydrothorax, can be distinguished from each other; while, in pneumothorax, it is confessed to be a source of almost unavoidable error. It is of no avail in diseases of the heart or great vessels; and even in phthisis is generally useless, and always proves insufficient to distinguish this from chronic pneumonia. Lastly, the integuments of the chest being œdematous, or fat, or flaccid, destroy the results.

*Auscultation* is defined by Dr. Stokes to mean an examination of the sounds which various causes "produce in the cavity of the chest:" this, however, is an incorrect limitation of the term, as auscultation expresses simply the act of examining by the ear, and has been applied to detecting the existence of fracture when the crepitus is indistinct, to ascertaining the existence of the fœtus in utero, and may possibly be applied to other purposes. The examination is seldom conducted by the direct application of the ear to the part, and, as some body is interposed, it has been called *mediate auscultation*,—rather a formal and affected appellation: the stethoscope is the instrument used; and, as most of our readers are aware, it consists of a cylindrical piece of wood, about a foot long, an inch and a half in diameter, bored longitudinally by a tube three lines in width; at one end is a conical excavation, supplied with a stopper. There is no mystery in making them, and they are now to be had at most of our surgical instrument makers. In using the instrument, we are directed to hold it as we do a writing pen, and to apply it flat upon the part, filling up with lint any inequalities, as from the projection of the ribs in a person much emaciated. When the force of the heart, or the phenomena of the voice, are to be examined, the instrument is to be furnished with its stopper; but this is to be removed when our scrutiny regards the respiration, or the sounds produced in certain diseases of the heart.

Before we attempt to recognise diseased conditions by this instrument, it is obviously necessary that we be acquainted with the phenomena attending a natural state of the functions: these accordingly are first described.

"The sound of respiration varies, first, according to the different parts of the chest examined; secondly, the frequency of respiration; and, thirdly, the particular conformation, the age or sex of the individual.

"When we apply the stethoscope to the chest of a healthy person, we hear, during respiration, a slight but very distinct murmur, which indicates the penetration of the air into the cells of the lung, and its expulsion.

"This murmur is nearly equally strong at every point of the chest, but especially where the lungs are nearest to the surface; that is to say, in the superior lateral and postero-inferior parts. The axilla, and the space comprised between the clavicle and edge of the trapezius, are the points where it is heard with the most intensity; over the larynx, the trachea, and root of the lungs, the respiratory murmur is distinctly heard; but it has a particular character, which causes us at once to perceive that the air is passing through a canal of greater diameter than the cells of the lung. In these situations we do not distinguish the expansion of the pulmonary tissue, and the air seems, during inspiration, to be drawn in through the cylinder,—during expiration, to issue from it. The sound of this respiration, which is called tracheal, may be exactly compared to that produced by a pair of bellows.

"The sound of respiration is more distinct as the latter is more frequent. A slow and deep inspiration is sometimes scarcely heard; hence it is often necessary to desire those whom we examine to breathe quickly and strongly.

"In children, women, and men of an irritable habit, the respiratory murmur is distinct and sonorous; the expansion of the cells is more perceptible, and the sensation is such, that they appear to be more dilated than in the lungs of a healthy man. This difference of sound is perceived most during inspiration. We find it also better marked as the person is younger. It generally remains until puberty, or a little beyond that age. In adults, the intensity of the respiratory murmur varies much; there are many healthy persons in whom it is scarcely heard, unless when they make a strong inspiration: in these cases the respiration is generally frequent. In some individuals, on the contrary, it is distinct, and even similar to that of infants; and these persons seem more disposed to diseases of the pulmonary organs." (P. 14—16.)

The author next enters at considerable length into a description of the pathological phenomena of respiration, in which its frequency and infrequency, quickness and slowness, regularity and irregularity, &c. &c. are minutely detailed. We take leave to pass this over, as not constituting the more immediate object of our present analysis, and take up the thread of the story again where the history of the stethoscope is resumed, as relating to the *respiratory murmur*.

"The sound or murmur of respiration may be stronger or weaker than in the natural state; altogether inaudible, or similar to that produced by the passage of the air through the trachea. It may be cavernous, as when the air passes into an excavation in the lung; and, lastly, it is heard combined with the different rales.

"When the respiratory murmur is stronger than in the natural state, it bears a great similarity to that of children; and on that account has been termed by M. Laennec, puerile respiration. This augmentation of the sound of respiration is not caused by any morbid alteration of the lung at the part where it is heard. It is observed in healthy parts, whose action is, as it were, increased for a time, in order to make up for that of the diseased portions.

"Puerile respiration is met with in one lung, when the other has lost its permeability, as from inflammation, tubercular development, &c. It is heard in pulmonary catarrh, after the re-appearance of the murmur of respiration, and in some cases of asthma and hysteria; but here it is combined with the most distressing dyspnœa. When a lung is but partially affected, puerile respiration is heard in the sound portions.

"The weakening or diminution of the respiratory murmur can only be ascertained by its examination at different parts of the chest, for it seldom happens that respiration is weakened in both lungs at once, or even in the entire of one. The intensity of murmur varies from the smallest diminution to the most complete nullity; its diminution may arise from many causes: thus it is produced by the incomplete obstruction of the minute bronchial ramifications, arising from thickening of their membranes, or the presence of mucus; it may occur also when there is an abundant crop of tubercles disseminated through the pulmonary tissue. We find it in pleurisy, while the false membranes are yet soft, and only beginning to be organised; and, lastly, it may arise from the diminished action of the thorax itself.

"The diseases in which we meet with absence of the respiratory murmur over a more or less considerable portion of the lung, are—pleurisy, accompanied by effusion; pneumonia, in its advanced stages; emphysema; pneumothorax; and pulmonary catarrh." (P. 24—26.)

From this quotation it will be perceived, that the respiratory murmur is either pure or combined with certain other sounds called *rales;* a French word for which we have no perfect synonym, although *rattle* comes nearer than any other. Adopting the term rale, however, as it is to a certain extent naturalised, we find it [redacted] to [redacted] mean[redacted] "any sound produced by the circulation of air through the bronchial tubes and pulmonary vesicles, differing from the natural respiratory murmur." Of these sounds there are four,—viz. the *crepitating*, the *mucous*, the *sonorous*, and the *hissing*.

The *crepitating rale* is compared to the crackling of salt when decrepitating, or that of a portion of dry lung when pressed between the fingers. It is supposed to depend upon increased determination to the air-cells, and thus becomes a pathognomic sign of the early stage of pneumonia; while, however, it likewise occurs in œdema of the lung, and in pulmonary apoplexy. In pneumonia, the rale does not at first conceal the respiratory murmur, but after a time, as the inflammation advances, it overpowers the natural sound of respiration. When the disease is about to terminate in resolution, the rale becomes more distinct, acquiring a humid character, while the respiratory returns; but, if the disease be running into hepatisation of the lung, the natural murmur is not resumed.

In œdema of the lung, the rale is analogous to the former, but less in degree; hence it has been called *sub*-crepitating.

In pulmonary apoplexy, the rale is present in circumscribed portions of the lung, while the natural sound is heard in the intervening spaces. After a time, the rale, from crepitating, becomes *mucous*, which leads us to speak of this modification of sound.

The *mucous rale* is best understood by the common illustration of the "rattles" in the throat of a dying person; this being the kind of sound, although, when confined to the minuter ramifications of the bronchiæ, it is much less intense. It appears to depend upon bubbles of air entangled in fluids of greater or less viscidity, and is closely allied to the crepitating rale; so much so, indeed, that Dr. Andral regards them as different varieties of the same sound, depending on their situation. Thus, we have a distinctly gurgling sound in the larger tubes; a crepitating sound in those which are minute; and something intermediate between the two in the intervening spaces. The mucous rale is characteristic of pulmonary catarrh in its advanced stage, and, in fact, of every disease of the lungs in which there is increased secretion from the mucous membrane of the bronchial tubes. It occurs in pneumonia in all its stages, and is extremely distinct (constituting the "gargouillement" of the French,) in phthisis, when softening of the tubercles has taken place. In pulmonary catarrh the rale is at first sonorous, and does not become mucous till the disease has made some progress; it is partial, unless the case be severe, when it may be heard over one entire lung. If present over the whole of both, the case is generally fatal.

Dr. Stokes, in the portion of his volume which follows, gives a very good description of the pathology of pneumonia; but our business is with the stethoscope; and, as this part of the subject is closed by the relation of two cases, "admirably" illustrative of its use, we shall insert them in full, with the author's comments, and then add a very few of our own.

"The following cases of pneumonia are admirably illustrative of the use of the stethoscope in this disease: indeed, in the second, without the use of this invaluable instrument, nothing could have been known about the true nature of the disease. The first, with its supplementary observations, is from the work of M. Andral, already quoted; the second formed the subject of an excellent thesis by M. Lenormand.

"Case I.—A carpenter, aged thirty-two, was seized with a violent shivering on the night of the 20th of April, 1822. On the morning of the 21st, he felt a pain first occupying the top of the left shoulder, *but which soon extended over the whole left side of the thorax; it was*

*augmented by coughing and by deep inspirations ; and, when he lay on the left side, it became insupportable. He had a dry cough, and sweating in the evening.* During the seven following days he kept his bed, and only took some emollient drinks. On the evening of the 27th he entered the hospital, was immediately bled ; and during the night was delirious. On the morning of the 28th, he had *short and hurried inspirations; frequent cough, with a considerable quantity of transparent, viscid, and sanguinolent expectoration.* The pain, less acute than on the preceding days, was felt, on percussion, over the left side from the axilla to the last ribs. He lay on his back. Upon percussion, the sound was dull, laterally and posteriorly, over nearly the whole of the inferior lobe of the lung. In this situation a slight crepitating rale was heard, without any mixture of the respiratory murmur. It was concluded, from these observations, that the inferior lobe of the left lung was partly engorged and partly hepatised.

" Pulse frequent, strong; skin hot and moist. The sweatings had continued every evening from the commencement of the disease. Tongue white; anorexia; thirst moderate; constipation.

" He was bled to twelve ounces, and thirty leeches applied over the left side. He had delirium during the night, which continued the next morning ; but the respiration was easier, the sputa were less bloody, and the crepitating rale much stronger and more extended ; seeming to announce that the hepatised portion of the lung was returning to the state of simple sanguineous infiltration. Less fever. As far as the pneumonia was concerned, the patient was evidently better; but the delirium proved a cerebral congestion, the more to be feared, as it should have diminished were it sympathetic with the thoracic affection. Enough of bleeding had been practised, as the patient was naturally of a weak constitution. Two blisters were applied to the legs, as revulsives at once from the head and chest. The delirium ceased towards evening, and did not again appear. On the next day he was in the same state. On the eleventh and twelfth days of his disease, the sound of the chest was less dull, and the crepitating rale more distinct. *The patient felt no more pain ; could make a deep inspiration easily ; the sputa, scarcely bloody, had become of the catarrhal character ; moderate fever ;* in a word, every thing proved that resolution was going forward. On the thirteenth day, a blister was placed on the left side of the thorax. During the fourteenth and fifteenth days, the natural respiratory murmur began to be heard, though still mixed with a crepitating rale. Sound of the chest no longer dull ; sputa catarrhal. On the sixteenth day, respiratory murmur more distinct, and only at intervals, and in some points, mixed with the crepitating rale. Pulse still a little frequent, but no other sign of fever. On the seventeenth day, the respiratory murmur was every where distinct and natural. Convalescence.

" Let us attend to the signs furnished in this case by auscultation and percussion. The sound was at first dull, and the crepitating rale feeble, without any mixture of respiratory murmur : from these signs it was inferred that hepatisation had already taken place. Further on, when the diminished fever, less dyspnœa, the catarrhal state of the sputa, the progressive return of the sound on percussion, all announced the resolution of the pneumonia: auscultation pointed out the stages of this desirable change, each day indicating the passage of the pulmonary tissue from the state of hepatisation to that of simple sanguineous infiltration. The greater or less intensity of the crepitating rale proved these different states of the lung, with an almost mathematical correctness. If this rale is very strong, without any mixture of the respiratory murmur, we may be certain that the whole of the lung where it is heard is only simply engorged, but that the sanguineous infiltration is very considerable. If the respiratory murmur is heard with the rale, the infiltration is less considerable, and much of the lung is still healthy. Sometimes the rale is heard only in insulated points, at long intervals, or even in a continued manner; but so feeble that it requires much attention and practice before it can be distinguished, and as it were separated from the surrounding murmur of respiration, which increases as the rale diminishes. In this case, the inflammatory action is very slight, or circumscribed.

"At other times, although the crepitating rale shall become more and more feeble, yet the respiratory murmur is not re-established: there is then a passage from the first to the second degree; a mixture of hepatisation and sanguineous infiltration. It is rarely that we find total absence of the crepitating rale, even where the hepatisation is considerable. In the latter case we have the tracheal respiration, and bronchophonia. When the portions of the hepatised lung become permeable to air, it is announced to us by the return of the crepitating rale, or by its greater intensity, if the latter has continued. It is a curious circumstance that, long after the other symptoms of pneumonia have disappeared, the respiratory murmur is still mixed with a little of the crepitating rale. What are we to conclude from this fact, but that the inflamed portions of lung generally return to their natural state in a much slower manner than could have been believed before the discovery of auscultation? Hence the liability to relapse in cases of pneumonia; hence also we may form some idea of the precautions necessary while this rale exists. If these are neglected, the disease, latent in its last periods, may return to the acute state; or, what is more common, the lung may pass into the state of chronic inflammation, terminating in a tubercular degeneration of this viscus, for which the patient may have been predisposed." (P. 45—50.)

In this case we have placed in italics those paragraphs which appear to us to have marked the nature of the disease, its increase in the first instance, and subsequent decline, in a manner so unequivocal, that no man would have thought it necessary to call in the assistance of the stethoscope, unless, like Dogberry, he was resolved "to spare no wisdom." Where is the practitioner so ignorant as to hesitate about the nature or treatment of a disease characterised by pain extending over the whole of the left side of the thorax, augmented by deep inspiration, accompanied by dry cough, and at night by delirium; all this supervening suddenly, and preceded by rigors. Suppose, however, (what for the sake of argument alone we can suppose,) that any doubt had existed with regard to the nature of the attack, let us observe the symptoms which presented themselves next day: he had now "short and hurried inspirations; frequent cough, with a considerable quantity of transparent, viscid, and sanguinolent expectoration." The pain was "less acute" than on the preceding day; but his pulse was still "frequent and strong." We cannot imagine any train of symptoms more distinctly characteristic of a severe inflammation of the lungs, relieved to a certain extent by the "sanguinolent expectoration," but not yet subdued.

Let us proceed. Next day, "the respiration was easier," the sputa "less bloody," and he had "less fever;" and, in a few days more, "the patient felt no more pain; could make a deep inspiration easily;" the expectoration had assumed a "catarrhal character," and the fever was "moderate." We repeat, that he who could not in these symptoms perceive the onset, full development, and gradual decline, of an attack of pneumonia, without the assistance of auscultation, mediate or immediate, deserves not that any confidence should be placed in his opinion, from what source soever it may be derived. Yet this case is given as "admirably illustrative of the use of the stethoscope."

We now come to the second case.

"The following is a remarkable case of acute *latent* pneumonia, accompanied by acute circumscribed *latent* pleurisy of the right side. The disease was promptly fatal, and was constantly marked by violent symptoms of inflammation of the mucous membrane of the intestinal canal, which, upon dissection, was found healthy.

"CASE II.—A man, aged sixty-one years, of a strong constitution, had enjoyed very good health until the 29th of November, 1823, when he accidentally breathed an irritating vapour, which was exhaled from a crucible containing silver in fusion. He immediately became drowsy, with a sense of weight in the head, and was obliged to quit his work. He continued in the same state till the 1st of December, when he had general sickness and nausea. On the 2d, he had frequent and violent efforts of vomiting. The pit of the stomach was painful. General illness much increased. *Respiration not at all laborious. He had neither cough nor pain in the chest.* He entered the hospital on the 3d, and on the 4th he had pain in the epigastrium, which was augmented on pressure. Headache. Abdomen hard; tongue red, and somewhat dry; great thirst; no appetite; frequent evacuations of a green colour. Cough rather frequent. Sputa liquid, yellowish, *not at all viscid; respiration apparently free*; *no pain in the chest.* Pulse frequent, full, but soft; urine very red. Sensation of weakness and pain in the lower extremities. Sound, on percussion, natural over the whole anterior part of the chest; posteriorly, it was dull over the three superior fourths of the right side. On the left, respiration was almost puerile; on the right side, it was only heard at the lowest part, and along the spine: over the remaining parts on the same side, nothing was heard during inspiration but a distinct crepitating rale.—Diagnosis: *Pneumonia in the first degree of nearly the whole right lung.*

"5th.—General prostration; delirium; respiration more freqnent; pulse intermittent. Abdomen hard, painful on pressure; sputa in small quantity, of the same character as before; sound of right side less clear; strong bronchophonia below the right clavicle, and posteriorly on the same side; crepitating rale very slight.—Diagnosis: *The pneumonia has made progress, especially towards the upper part of the lung.*

"At four o'clock that day, general debility increased; abundant and involuntary dejections; comatose delirium. Bronchial respiration anteriorly on the superior third of the right side, under the axilla, and posteriorly. The crepitating rale has disappeared.—Diagnosis: *Hepatisation of the superior part of the right lung.*

"6th.—Stools less abundant during the night. Much delirium till five o'clock in the morning; none at the hour of visit. Intense thirst, which has continued during the night; tongue soft, more humid, and less red. Abdomen still swollen, but more soft, and not painful. Respiration more accelerated, and accompanied by a tracheal rale; little cough; expectoration trifling. No bronchial respiration; over the whole right side, the respiratory murmur is null; on the left, always puerile.

"At four P.M.—Extreme prostration; tracheal rale very distinct; pulse small and frequent; tongue humid, soft, and slightly foul; intense thirst; epigastrium very painful on pressure. Vomiting has ceased. Constipation. Intellectual faculties entire.

"7th.—Augmentation of all the symptoms. Epigastrium continues painful; tongue pale and very humid; pulse hardly sensible. Died at eleven A.M.

"*Dissection.*—The right lung voluminous and in one mass, was almost covered by the costal pleura, which, adhering intimately to the lung, had been torn out with it, and preserved the impression of the ribs, though the pulmonary tissue presented no such appearance, as was proved by dissection. The two pleuræ, united throughout the whole of their extent, presented a cavity at the superior part of the lung, capable of containing a hen's egg. The parietes of this excavation were formed by the two pleuræ, and it was filled with a liquid brownish pus, which, coming from the lung, had passed into this abscess, in consequence of an erosion of the pleura pulmonalis. A similar purulent collection existed between the superior and middle lobe. The superior third of the lung was compact, and infiltrated with a semi-concrete pus, which in many cases formed abscesses capable of containing a nut, many of which communicated with one another. The middle part presented the red hepatisation, and the inferior a strong sanguineous congestion. These three states were divided by well-marked lines of demarcation, corresponding to the interlobular fissures.

"The left lung was for the most part healthy, except at its posterior part, where it was inflamed to the first degree; the inferior lobe was beginning to pass into red hepatisation. The heart was natural; its right ventricle containing a very adherent fibrinous concretion.

"The abdominal viscera presented their natural aspect. The stomach was remarkably flaccid; its mucous membrane in some parts slightly injected, and towards the pylorus of a light red colour. The whole of the intestinal tube being opened, the end of the jejunum only was found with a vascular injection. The other viscera were healthy. Pancreas a little harder than natural." (P. 50—55.)

This case was one of considerable obscurity, and the author has placed in italics, passages in which we have retained the same character: they are obviously intended to mark certain symptoms, as leading to the belief that the disease was not thoracic. We have to observe, however, that the first and most striking part of the account relates to the state of the patient before he applied at the hospital; and, as it is not stated that he had been seen by the reporter, we think it fair to infer that he had not; consequently the same confidence cannot be placed in the accuracy of the narration. Be this as it may; he entered the hospital on the 3d,—in what state we are not informed; but next day, when the first authentic report is given, he had "rather frequent cough," with "liquid, yellowish" sputa, and a "frequent, full, but soft" pulse, while his urine was "very red;" and, when we consider that these symptoms had been brought on by breathing "an irritating vapour," we do not think it required any extraordinary sagacity to conjecture that the lungs might be inflamed. We are not, however, disposed to deny that there was a good deal of ambiguity about the case. Let us observe, however, the result afforded by the stethoscope under these circumstances. The disease was pronounced to be "pneumonia in the first degree of nearly the whole right lung;" then we are told that "the pneumonia has made progress, especially towards the upper part of the lung;" and, lastly, that "hepatisation of the superior part of the right lung" had occurred. So says the mediate auscultation. But, when we turn to the dissection, we find that, instead of the upper part of the lung being simply hepatised, "the two pleuræ, united throughout the whole extent, *presented a cavity at the superior part of the lung capable of containing a hen's egg*." This excavation, we are told, was filled with pus, which had come from the lung through an erosion in the pleura pulmonalis. Here, then, was a large abscess between the two pleuræ, communicating with the lung by ulceration,—*the presence of which was not detected by the stethoscope.* "A similar purulent collection existed between the superior and middle lobe." Here, then, is a *second large abscess not detected by the stethoscope.* Again, "the superior third of the lung was compact, and infiltrated with a semiconcrete pus, which in many places formed abscesses capable of containing a nut, many of which communicated with one another." *Here, then, is a whole set of abscesses, which were not detected by the stethoscope.* We really must confess ourselves at a loss, therefore, to discover in what respect this case, any more than the preceding, is "admirably illustrative" of the use of this instrument.

‡ *Sonorous rale.*—The comparisons used to convey an idea of this sound are somewhat incongruous: it is likened to the "snoring of a person asleep," the tone "of the bass-string of a violin," and to "the cooing of turtle." It is supposed to arise from narrowing of the bronchial tubes, caused by determination of blood to the mucous membrane, or from some other change in the form of these canals. We are told that—

"We must be careful not to confound this rale with the guttural sound produced during sleep: the first has its seat in the chest, and is not heard by the naked ear; the second, on the contrary, is solely derived from the manner in which the air inspired and expired strikes the velum of the palate. By means of the stethoscope, it is easy to perceive that it does not take place in the cavity of the chest.

"The sonorous rale is the pathognomonic sign of acute bronchitis.

"In pneumonia, accompanied by bronchitis, we have the sonorous and crepitating rales complicated. In the dry pulmonary catarrh, or asthma, the sonorous and hissing rales are met combined. The first varies little; the second is of great mobility, disappearing for a greater or less time, in consequence of coughing, or without any perceptible cause; and then returning suddenly, and with a different intensity. Sometimes both are constant, distinct, and accompanying the greatest part of the organ. The catarrh is then extensive and violent.

"In the humid variety, the same phenomena may exist, but ordinarily they are complicated with a third, namely the mucous rale, which becomes entirely predominant after the acute stage is past, and thus characterises the disease." (P. 68, 69.)

*Hissing rale.*—This is a prolonged wheezing sound, accompanying either the end or commencement of inspiration or expiration: it is compared to the "cry of young birds," the sound of "two pieces of oiled marble suddenly separated," or that of a "small valve" in action.

"The hissing rale is owing to the presence of a scanty but viscid mucus, obstructing more or less completely the small bronchial ramifications, through which the air is obliged to pass before it reaches the vesicles. When it is heard over a considerable portion of the lung, respiration is very laborious. It is during the existence of this rale, that we observe the sputa presenting an arborescent appearance, resembling the form, calibre, and ramifications of the minute bronchial tubes, from which they have been expelled by the efforts of coughing.

"The principal affections in which the hissing rale is heard, are emphysema of the lungs and the chronic pituitous catarrh of M. Laennec.

In the acute species of catarrh, it occurs complicated with the sonorous and mucous rales.

"In emphysema, the respiration is not heard over the affected part, while the chest sounds well, or even louder than natural, on percussion. A slight hissing rale is heard from time to time, at the points corresponding to the affected part." (P. 78, 79.)

Having thus dismissed the rales, we next come to the *phenomena of the voice,* which are either natural or pathological. The former consist of a certain resounding of the voice, which produces over the whole of the chest a trembling or vibration, capable of being produced by the application of the hand. When the stethoscope is used, a confused reverberation of the voice is heard, varying in intensity according to the point examined. It is most distinct in the axilla, and between the vertebral column and the edge of the scapula, and about the angle formed by the clavicle and sternum. In other regions of the thorax, particularly the lower and back parts, it appears weaker, giving a confused inarticulate sound. In deep-toned voices, the resounding is stronger, but more confused; in shriller voices, as those of women and children, it is much more distinct. It is, of course, necessary to study the natural phenomena in the first instance; and, being familiar with them, we have the better chance of catching (if we may so call it) the deviations from those which indicate disease. These are referred to four varieties—Bronchophonia, Pectoriloquism, the Metallic Tinkling, and Egophonia.

*Bronchophonia* is the name given to a vibratory sound of the voice, when it is louder than natural, or occurs at a part of the chest wherein it is not heard during health. It does not form an articulate sound, but is confused, and seems barely "to enter the bottom of the stethoscope, without traversing the tube to arrive at the ear of the observer." This modification of sound is supposed to depend upon induration of the lungs, produced by inflammation, or by a tubercular mass; a medium being thus formed which is better fitted for transmitting the murmur of the voice. The best method of obtaining an accurate idea of this phenomena is stated to be by applying the instrument to the point of the chest corresponding to the root of the lung, while the patient is speaking. When dependent upon extensive ulceration of the pulmonary tissue, it is always accompanied by bronchial or tracheal respiration. This symptom is chiefly of use by enabling the pathological inquirer to institute a comparison between the two sides of the chest, and as an additional proof when coexistent with other phenomena.

The next modification of sound being one regarded as very important, we shall give the author's account of it without abridgment.

"*Of Pectoriloquism.*—We say that a patient has pectoriloquism, when the voice, distinctly articulate, seems to issue directly from the place where the stethoscope is applied, and to traverse the canal of that instrument.

"Pectoriloquism is either perfect, imperfect, or doubtful. It is *perfect,* when the articulate and well-defined voice traverses the cylinder, and arrives at the ear with its natural or an increased intensity of sound. It is *imperfect,* when the articulate voice reverberates strongly under the stethoscope, appearing to approach the ear, without, however, traversing the entire tube. It is *doubtful,* when the voice appears sharp and restrained, like that of ventriloquists; not traversing the tube, and approaching to mere bronchophonia. Imperfect and doubtful pectoriloquism can only be trusted to as indicative of organic lesion, when they exist on one side only, or when they coexist with other symptoms observed by examining the respiration.

"The most perfect pectoriloquism may sometimes take on the characters of the imperfect, or even doubtful species, for a short time. It may disappear from time to time, becoming thus intermittent. This change shall be explained, after the exposition of the causes of pectoriloquism.

"This phenomena is owing to the presence of excavations in the lung, however produced, communicating freely with the bronchial tubes, and either in part or completely empty. Pectoriloquism may be met with in all parts of the chest; but it is most frequently observed in the axilla, the space between the clavicle and the trapezius, that immediately under the clavicle; and the infra and superior spinous fossæ. These all correspond to the superior part of the lung; and it is here that the excavations produced by the softening of tubercles are most frequently observed. Pectoriloquism varies with the sound of the voice, the size of the excavations, their form, and the density of their parietes, the adhesion of the two pleuræ over these cavities, and the facility or difficulty with which the air enters them.

"The more acute the voice, the more evident is pectoriloquism; in persons with a deep voice, it is almost always imperfect, and sometimes doubtful. Aphonia does not cause it to disappear completely, and it often happens that we can distinguish better what the patient says by means of the stethoscope applied over the excavation, than with the naked ear at the same distance.

"In order that pectoriloquism may be perfect, it is necessary that the excavation be only of a moderate size. In very large excavations, pectoriloquism is changed into a deep sound, analogous to that of the voice transmitted to some distance through a trumpet or cone of paper. Where, on the contrary, the cavities are very small, it is frequently doubtful, especially if the excavation is situated in the centre of the lung, and surrounded by parts still easily permeable to the air.

"The irregularity, or the direct communication of a number of cavities with one another, causes pectoriloquism to appear somewhat stifled and confused; the voice appears badly articulated. The firmer and thinner the parietes of the excavations, the more perfect is pectoriloquism. When, by a process of cicatrisation, a fibro-cartilaginous membrane is formed over the entire surface of one of these cavities, the pectoriloquism acquires a metallic tone, sometimes so considerable as to hinder our accurate perception of the sounds.

"An excavation situated at the surface of the lung, and whose thin parietes do not adhere to the costal pleura, but collapse during expiration, does not cause pectoriloquism. On the contrary, a superficial excavation, with thin adherent walls, gives so strong a pectoriloquism as to fatigue the ear.

"This phenomenon is more evident in proportion as the cavity contains less fluid, because the bronchial communication is then generally free, permitting an easy access to the air. This communication, however, may be destroyed, more or less completely, by the accumulation of the sputa in the bronchial tubes: this renders perfect pectoriloquism doubtful, and gives it that intermittent character which is not unfrequently observed. It may be often remarked, when pectoriloquism is absent in a patient in whom we have observed it but the evening before, that the expectoration has been scanty, or almost entirely wanting.

"True pectoriloquism is heard in the affection termed by M. Lænnec *dilatation of the bronchial tubes.* Of this he has given a case, art. 149 of his great work, (*De l'Auscultation Médiate.*)

"A woman labouring for some years under habitual yellow expectoration, was evidently pectoriloquous on the right side above the third rib. Upon dissection, two bronchial tubes, dilated to three times their natural size, were found in the corresponding part of the lung; one of them terminated in a sort of cul-de-sac, large enough to contain a small nut.

"M. Andral has given a very instructive case of dilatation of the bronchial tubes, giving rise to pectoriloquism.

"A middle-aged man entered the hospital of La Charité, labouring under the symptoms of pulmonary consumption. The respiratory murmur was scarcely heard on the left side of the chest, while anteriorly, on a level with the heart, and posteriorly, below the inferior angle of the scapula, evident pectoriloquism was observed. He sunk, after remaining nearly two months in the hospital. The following is the account of the dissection, in the words of M. Andral.

"The left lung generally crepitated but little; it, however, floated when plunged in water. In the superior lobe there existed a cavity large enough to contain a middle-sized nut, and filled with a fluid analogous to the matter of expectoration. A bronchial tube, as large as a writing pen, opened into it. Dissection soon convinced us that its parietes were continuous with those of the cavity itself, forming the same tissue. We found in both the mucous membrane red and thickened, and the fibrous membrane, with some traces of the cartilaginous rings. It was now very evident that what we had taken at first for a tuberculous excavation, was nothing but a considerable dilatation of a bronchial tube. In many points of the parietes of the dilated portion, small orifices opened, which led into other bronchial tubes." (P. 88—93.)

*Metallic tinkling.*—This sound resembles the falling of a drop of water into a deep vessel, of a grain of sand into a glass cup, or the sound emitted by a vessel of metal or porcelain when struck with a pin; it is of short duration, and is heard on raising the patient, or causing him to cough; it may be occasionally perceived when he merely breathes or speaks, but not nearly so well as when he coughs. When it is present along with pectoriloquism, the two sounds are heard traversing the tube of the stethoscope together. Where, however, the pectoriloquism is not coexistent, the metallic tinkling is heard within the chest, and compared to the sound of a wire struck with the finger.

"As this peculiar sound depends upon the vibration of the air caused by respiration, the voice, or coughing, on the surface of a liquid partly filling an unnatural cavity in the chest, it can only exist in two cases: first, where a serous or purulent effusion coexists with pneumothorax, arising from a fistulous opening into the cavity of the pleura; and, secondly, where a large excavation, half filled with fluid pus, occurs in the substance of the lung.

"In order that it shall happen in the first case, it is necessary that a fistulous opening be found between the cavity of the pleura and some of the bronchial tubes: thus it becomes a sign of this triple lesion. The distinctness of the sound is in proportion to the diameter of the fistulous opening, and the extent of the vibrations teaches us the space occupied by air. It is in general stronger as the quantity of air existing in the chest is greater; and hence we may conclude, when it is indistinct, that the liquid effusion is considerable, and vice versâ.

"When it arises from the vibrations of the voice, or from coughing, acting on the surface of puriform matter in a large excavation of the lung, it presents some important differences. Its indistinctness, and the small extent of its vibrations, teach us that it occurs in a very circumscribed space; it appears to enter the cylinder, and is combined with pectoriloquism, which, with the other symptoms, enables us easily to distinguish this from the former case." (P. 104—106.)

*Egophonia* consist in a strong reverberation of the voice, which appears shrill, interrupted, and "quivering like that of a goat." It most commonly occurs between the spinal column and the internal edge of the scapula, but it may likewise exist over the whole extent of the chest. Egophonia is thus heard over a much more extended space than pectoriloquism; and it is stated always to indicate the presence of a small quantity of liquid in the cavity of the pleura, or of false membranes in a soft state. This last assertion rests on the authority of M. Collin. When, however, the effusion is either much increased or diminished in quantity, this symptom disappears. The points where egophonia is most commonly heard, are those which correspond to the upper portion of the effused fluid. If the patient lies on his belly, it is either not heard at all, or at best but very feebly in the space between the spine and scapulæ; while it is still perceived in the side. If the patient lies on the side opposite the seat of disease, this sound is rendered less apparent: it is heard to most advantage when he lies on his back or sits up. Even those best skilled in the use of the stethoscope appear liable to be deceived by this sound. "It has frequently happened (says M. Andral,) that, after having believed that egophonia, and other signs indicative of effusion, existed, we have discovered our error from the examination of the opposite side."* Of course we ought, therefore, always to examine both sides before we draw any conclusion. It further appears that the egophonia frequently occurs only at intervals, or in the pronunciation of certain words: thus M. Andral has known a patient in whom this sound was only present when he articulated the word "*oui.*" —Credat Judæus!

[To be continued.]

### DIVISION II.

### FOREIGN.

ART. II.—*Nuovo Metodo di Curare la Trichiasis.* Memoria del Professore A. VACCA BERLINGHIERI. (From the *Annali Universali di Medecina.)*

*New Method of curing the Trichiasis.* A Memoir by Professor A. VACCA BERLINGHIERI.

THIS Memoir, first published in the "Nuova Giornale di Litterati," a work not exclusively devoted to medical or surgical subjects, has since been reprinted in various other periodical publications; and, considering the conflicting opinions that have been propagated and maintained relative to the operation of which it treats, and the celebrity of the author, we conceive that we cannot perform a more acceptable service to our readers than that of presenting them with the details. This Memoir, in its original state, occupies a very considerable space; for M. VACCA not only gives a description of the different species of trichiasis, as described in systematic writers, but explains at some length, and comments upon, the means hitherto put in practice for the purpose of remedying them. We shall not follow this plan exactly, but shall limit ourselves (after saying a few words relative to the disease,) to a description of the means of cure, as recommended by the illustrious author.

After a page or two of preliminary remarks, M. Vacca says—

It is well known that oculists, in general, admit of three different species of trichiasis: in the first the hairs are turned inwards, and with them the tarsus, in a manner more or less marked, either in one point only or throughout the whole extent of its free margin. In the second species, the tarsus preserves its proper direction perfectly, and the hairs

* *Clinique Médicale,* tome ii.

are turned against the eye. In the third, both the cartilage and the hairs preserve their natural direction, but there is a preternatural row of hairs, which are either altogether or in part turned inwards against the ball of the eye. The two first species are generally admitted, but the third is controverted; names of equal respectability maintain the opposite sides of the question.

M. Vacca, after giving an epitome of the opinions of the principal oculists as to the causes of these different species of deformity, proceeds to enumerate the methods which have been adopted for remedying it, commencing with the plan recommended by Schreger in Germany, and terminating with Guthrie's modification of Crampton's operation. Upon each of these plans he afterwards comments with the acumen of a practised and experienced master, and finally concludes by rejecting them all, as either partially or wholly inefficacious, or as substituting a deformity as intolerable as the original disease. Towards the termination of the above criticism, our author uses the following language.

From this exposition it results that surgery possesses means of cure in that species of trichiasis in which the tarsal cartilage is slightly inverted together with the hairs, and that not only without producing much pain, without leaving behind it any deformity, nor producing any imperfection in the organ of vision; secondly, that art can produce much amelioration in that species which is formed by complete and total inversion of the cartilage throughout its whole extent, by means of an operation, not only extremely painful, but which leaves a constant deformity, and deprives the eyelids ever after of its lashes; thirdly, that there is no method known of curing that kind of trichiasis in which some single hairs, or some groups of hair, either naturally existing or of new formation, are directed against the globe of the eye, in which the tarsus remains in its situation, or is but slightly inverted, since all the methods hitherto put in practice either fail in overcoming the disease, or convert it into another of no less consequence. Nevertheless, (continues M. Vacca,) the mode of remedying this deformity is not attended with any great difficulty; and, if this desirable end has not hitherto been attained, it is because the ancient surgeons, from the want of accurate anatomical knowledge, have not pursued the proper course, and the moderns have been contented to follow their track. The bulbs of the hairs of the eyelids are situated, as every one knows, by the side of each other, disposed in a line upon the external face of the free margin of the eyelid, involved in cellular substance, and covered solely by a thin integument.

To cut this integument,—to uncover the bulbs of the inverted hairs,—to extirpate or to destroy them, is the method of cure proposed by the author. This method, considered theoretically, appears infallible in its results; and such was the impression it made upon M. Vacca's imagination when he first conceived the project, but he did not choose to publish it until it had been verified by actual observation. " I might be permitted (says our author,) to dispense with an account of this operation, it being easy for every surgeon to conceive the mode of performing it: nevertheless, I will describe it, (he adds,) in order to spare some the trouble of meditating upon it, and to offer to others the means of proposing useful modifications.

To perform this operation more readily, it is necessary to be provided with an instrument which M. Vacca calls a spoon, a small knife, a very fine pair of dissecting forceps, and a small pair of scissars. The two first instruments not being well known, we have annexed a sketch of them, in order to save a tedious and imperfect description.

*Fig: 1.*

*Fig: 2.*

Fig. 1, represents the spoon of its proper dimensions. A. This part of the instrument, made of tortoise-shell, of horn, or ivory, has two faces, one slightly convex, the other slightly concave. B, C. The two extremities. C contains a little groove, D, D, D. The extremity B is firmly fixed into a suspension of the eyelid, made of silver wire, and which may serve also as a handle.

Fig. 2, represents the knife. The edge A is very sharp; the side B is blunt.

The patient being properly placed in a chair, the face turned towards the light, an assistant places himself behind the patient, his breast presenting a firm rest for the head of the person operated on, as in the operation for cataract. The operator, placed immediately in front of his patient, either standing or sitting, as may be most convenient to him, raises up the eyelid, and ascertains the number of hairs that are inverted, and the extent which they occupy. Having done this, he traces with a pen and ink a line upon the integument covering the eyelid, parallel to its free margin, and a quarter of a line distant from it; and this line is to be extended in length, so as to show with precision, upon the surface of the lid, the space which the misdirected lashes occupy on the internal surface. He then introduces the spoon between the palpebræ and the globe of the eye, in such a manner that the free margin of the former is placed in the groove which is situated on the convex surface of the instrument. He then draws this from the globe of the eye as much as possible, to prevent it from being irritated, and to extend the palpebra more thoroughly. Having done this, he confides the spoon to an assistant, who with his right hand, if the eye operated upon is the right eye, (if not, with his left,) draws, distends, and fixes the palpebra upon the spoon, by means of the index and middle finger placed upon the angles of the eyelid, so as to leave the part to be operated upon exposed and free. With the other hand passed under the patient's chin, he will hold the handle of the spoon, taking care to maintain it in the position in which it was delivered to him by the operator. Things being so disposed, the surgeon makes, with the knife above delineated, two small vertical incisions, which are to commence a line and a half above the free margin, and are to terminate precisely at that point. These two parallel incisions include exactly that space which is marked out by the inked line, and are only to include the integument. The lateral incisions being finished, a third transverse one is to be made beneath the line marked upon the palpebra, and parallel to it. This is to unite the two lateral incisions, and is also to include the skin only. A flap being thus formed, it is to be turned back, taking hold of it either with the forceps or between the nails, and dissecting it with the knife from the parts beneath. The flap being turned back, the bulbs present themselves; but it is not always easy to perceive them clearly, and to remove them, because not only does the blood contribute to conceal them, but the fixed cellular membrane that surrounds them does not render it very easy to lay hold of them. On this account the surgeon must clean the wound thoroughly from the blood, and be provided with a very fine and excellent pair of pincers, and with these, and the knife, or with a small pair of forceps, take away all that are found between the inverted skin and the external face of the free margin of the eyelid. That being done, the operation is finished; and the surgeon reapplying the flap of integument to its natural position, keeps it easily in its situation by means of adhesive plaster, without the necessity of any other dressing.

It is necessary to observe, that, if the inverted lashes are at a distance from one another, and in the interval between them there are many of the hairs having a natural and proper direction, it will be advisable only to destroy the bulbs of the former, without disturbing those of the latter. It is not necessary to discuss the modifications which this operation requires when performed upon the lower eyelid: the experienced surgeon can readily adopt his means to the different circumstances of the two cases.

Although M. Vacca succeeded perfectly in the two first cases in which he adopted this method of operating, nevertheless he felt that the extirpation of the bulbs might easily confuse a person not accustomed to delicate operations, and therefore he was anxious to render it more easy of execution, and consequently more manageable to every surgeon; for this purpose, in the third case, besides the ordinary apparatus, he prepared a kind of wooden probe, with a small quantity of cotton thread at the extremity. The operation was commenced in the ordinary manner; but, as soon as the flap was raised up, instead of trying to lay hold of the bulbs with the pincers and to extirpate them, he touched them with the armed end of the probe dipped in nitrous acid. It is scarcely necessary to observe, that the cotton must not be so much impregnated with the acid as to let it run upon the neighbouring parts. This plan, which undoubtedly renders the operation both easier as well as quicker, although not less painful, appears to have an equally successful result.

With respect to the lashes themselves, the bulbs of which have been destroyed or extirpated, two methods may be followed,—either to take them away, or to let them fall off spontaneously. This latter circumstance takes place sometimes sooner, at other times later, but not before the sixth day. Neither is it always advisable to suffer the lashes to fall off, since, on account of the extreme sensibility of the parts, their presence occasionally produces great inconvenience, and therefore it becomes necessary to extirpate them directly.

The method described, observes our author, appears to unite all the advantages hitherto sought for in vain. By means of it the bulbs are absolutely destroyed, and consequently the lashes; it does not change the direction of the free margin of the palpebra, nor does it interrupt its continuity; therefore, the weeping of the eye, the ingress of air and light during repose, and the introduction of foreign bodies, are all prevented. It does not produce deformity; and the inutility of all apparatus and dressing after the operation, excepting some strips of sticking plaster, may be reckoned as something in favour of this operation.

Our author illustrates the above details by the relation of three cases, which we shall present to our readers; passing by a page or two of observations on the usual means of procuring union of the parts, after the common operation for the cure of the disease. These remarks would lengthen our review too much, and have no immediate reference to the principal subject of this Essay; and therefore we proceed at once to the detail of the three cases, in which this new mode of operating was put in practice.

"CASE I.—Rosa Marracini, of Pontedera, twenty-one years of age, had been afflicted for a long time with chronic ophthalmia, produced by that species of trichiasis which is accompanied by an inversion of the eyelid, and from which inconvenience she had been relieved by the destruction of that part of the integument covering it. Not long after her cure, the trichiasis reappeared; but the tarsus had not again abandoned its natural direction, although a few of the hairs had left their usual situation, and directed themselves against the globe of the eye. M.

Vacca, perceiving the inconvenience of repeating the former operation in this case, operated upon the patient in the hospital according to the new method. The operation proved very painful. No other dressing was placed upon the eye but a bandage and a light compress, to exclude the air. The pain, which was rather severe for several hours, was mitigated by a dose of laudanum. Notwithstanding this, the eyelid swelled, inflammation was excited, and suppuration was established in the wound on the fourth day. On the sixth day, the hairs that were directed inwards fell off spontaneously; the chronic ophthalmia gave way in a short time, and the wound was cicatrised by the twelfth day; and a month afterwards the patient left the hospital perfectly cured.—This case is related by a surgeon, named GAMBERRI.

CASE II.—Maria Gallizea, of St. Sisto al Pino, of a feeble constitution, twenty-one years of age, and a cook by profession, was affected, in consequence of chronic ophthalmia, with trichiasis, complicated with inversion of the tarsal cartilages of the upper eyelids. Subjected in the hospital to the common operation for this complaint, she was entirely cured; but the cure was not permanent: a fresh attack of ophthalmia supervened, and a surgeon, observing that some of the hairs of the eyelids rubbed against the eye, extirpated them. This extirpation produced, as usual, temporary relief; but the patient at length, weary of repeated relapses, presented herself again at the hospital in the month of March, 1825, to consult M. Vacca. On examining the state of the eyelids, he found that the cartilage of the right eye maintained its proper direction, but that one solitary hair, which appeared to be fixed in the interior face of the free margin of the lid, was the only cause of the irritation of the globe of the eye. On the left side, a slight and partial inversion of the cartilage was found. To correct this the common operation only was necessary; but, to overcome the defect which existed on the right side, the Professor, employing the means above detailed, laid bare the bulb of the inverted hair, and destroyed it. The blood which flowed from this little incision rendered the destruction of the bulb rather troublesome. The wound being dressed, the patient soon became easy; no inflammation ensued, and union by the first intention took place. At this period the hair had not fallen off, and it was left in its situation, in order to see what would occur. On the sixth day it fell off, and the chronic ophthalmia, which it had kept up, disappeared. The girl was kept in the hospital until the 10th of May, and at that time she quitted it, perfectly cured. On the 10th of June she went, at the request of the surgeons, to the hospital, in order that the eye might be examined; and it was found, not only that the hair had not reappeared, but that a certain depression, or little pit, existed in the spot where the bulb had been; and that no hair, excepting the inverted one, had fallen from the lid.—This case is signed by D. GARGANI.

CASE III.—Leopold Sforzi, of Pisa, thirty-five years of age, of a good constitution, was attacked, in the year 1814, with a violently acute ophthalmia, which terminated in an abscess in the globe of the right eye, and in chronic ophthalmia of the left, which at length produced the inversion of the cartilage of the upper eyelid, and consequently of the lashes implanted in it. In 1823, this man presented himself at the hospital. The common operation for trichiasis was performed, and for a time the cure appeared to be complete. Some months afterwards, however, the chronic ophthalmia reappeared, and the man returned to consult Professor Vacca, who found that the cartilage maintained its proper direction, but that three hairs, rather longer and larger than ordinary, having abandoned their natural position, rubbed against the eye. The patient would not at that time submit to an operation, but preferred the extirpation of the hairs, which was repeated from time to time; and he did not come back to the hospital till the 24th of April, 1825, the eye then being in a high state of inflammation, attended with an obvious opacity of the cornea. M. Vacca now persuaded the patient to submit to his new operation, and, having laid bare the bulbs, he destroyed them by means of the nitric acid. This rendered the operation both extremely short and easy, but it was not less painful. The pain, however, soon yielded, and a very trifling degree of inflammation and suppuration succeeded. On the fifth day, the hairs not having fallen off, and continuing to produce great inconvenience to the patient, the Professor extirpated them with the forceps: the irritation of the eye then disappeared. On the sixth day, the little wound resulting from the operation was healed, and the hairs, which, after common extirpation, were usually renewed at the end of eight or ten days, had not shown themselves at the end of two months; but a small depression was, as usual, observed in the situation of the bulbs that had been destroyed. The complete deprivation of the hairs from that portion of the palpebral margin which suffered from the action of the caustic, must also be noted. For forty days all the lashes remained in their proper situation, and every one was surprised that the operator had been so fortunate as only to destroy the bulbs of the inverted hairs; but, at that epoch, they began to fall off one after the other, and the surprise ceased.

---

ART. III.—*Recherches Anatomico-Pathologiques sur la Phthisie.* Par P. C. A. LOUIS, Docteur en Medicine des Facultés de Paris et de St. Petersbourg; Membre adjoint de l'Academie Royale de Medicine de Paris, Correspondant de celle de Marseille. *Precedées du Rapport fait a l'Academie Royale de Medecine,* par MM. BOURDOIS, ROYUR, COLLARD, and CHOMEL.—Pp. xxiv. 560. Paris: chez Gabon et Ce. 1825.

*Anatomical and Pathological Researches concerning Phthisis.* By P. CH. A. LOUIS, Doctor of Medicine of the Faculties of Paris and St. Petersburg, &c.

[Concluded from page 259.]

HAVING, in a previous article, given a tolerably extended account of the pathological anatomy of Phthisis, we now enter on an examination of the second part of M. LOUIS's work, containing a detail of the symptoms of that disease, together with its principal complications and causes, terminating with a few reflections on the method of treatment.

Without further preface, we proceed to the first chapter, which commences with what our author calls a general description of the complaint. Following the example of M. Laennec, our author divides the course of phthisis into two periods; the one anterior, the other posterior, to the softening and evacuation of the tubercular matter through the bronchiæ.

*First epoch.*—In the greater part of the cases, the disease began without any known cause. A third of the patients attributed its invasion to alternations of heat and cold, to which they were exposed in the exercise of their different occupations, or other similar causes; but of these, many did not speak of this in a very positive manner. A very small proportion of patients fixed the origin of the catarrh, with something like precision, to twenty-four, thirty-six, or forty-eight hours, from the application of the cause to which they ascribed it. The commencement of the disease was generally by a trifling cough, to which no attention was paid, being generally attributed to the effects of a common cold. The expectoration was usually clear, and resembling broken-down saliva: in a tenth of the subjects, however, the cough was dry for a period of one or more months. Sometimes it took place by fits, and made rapid progress. After the lapse of a greater or less period of time, the sputa were more opaque and greenish; in the second period, they entirely changed their aspect. Sometimes hæmoptysis preceded the attack, but more generally it was a consecutive symptom. The respiration was not sensibly affected at first, and dyspnœa did not become troublesome, in a certain number of subjects, at a more advanced period of the malady. In a considerable proportion of cases, severe pains between the shoulders, and at the sides of the chest, appeared after the commencement of the disease. If auscultation was practised at this period of the complaint, the respiratory sound did not appear to be altered; at least, with few exceptions: in these, respiration appeared feeble under one or both clavicles, or else there was in the same points, and in a limited space, a little sonorous and mucous rattle, and the chest rendered a sound somewhat less clear than the opposite side. Besides these local symptoms, others of a more general nature prevailed: sometimes alternate chills and heats, or nocturnal perspirations, took place from the very beginning. With few exceptions, the appetite was undiminished for some time; afterwards it fell off gradually. Sickness after eating occasionally took place, if the cough was violent; purging very seldom. Emaciation appeared soon after the beginning of the disease, and increased slowly.

*Second epoch.*—At this time the cough was usually more troublesome and frequent; the expectorated matter was greenish, striated with yellow lines, opaque; it was of a round form, contained no acid, and was (as it were) lacerated. Sometimes, under the influence of medicine, it lost this character for a short time; and it was sometimes accompanied by an expectoration similar to that met with in the first period. Pain was increased at this time, and spitting of blood frequent, though seldom to any great extent. Pleuritic symptoms, of great intensity, occasionally supervened. The patients generally stooped the head. Their mode of lying in bed was various, but generally on the side opposite to the large excavations. Auscultation did not appear, from our author's description, to possess any great advantage over simple percussion in these cases, as, in one-third of the subjects, this latter produced no sound for a considerable extent below one or both clavicles.

With regard to the hectic fever and diarrhœa, M. Louis observes nothing that our readers are not familiar with.

With respect to the duration of the disease, the following are the results of our author's researches:—Out of 114 cases of phthisis, the duration of which had been observed with the greatest possible exactness, rather more than two-tenths died from the first to the sixth month; four-tenths from the sixth to the twelfth; a little less than a fourth from one to two years; and rather less than a fifth from the second to the twentieth year.

With regard to the influence of sex, it appeared that, when death ensued within a year, the proportion of females to males was as thirty to forty-two.

The mortality from phthisis, compared with all other maladies, was as one to two: that is to say, of 358 subjects who died in M. Chomel's wards in the space of three years and a half, 123 perished from phthisis; and if to this number may be added those who, though affected with tubercles, died of other complaints, it brings the proportion of those who died with consumptive symptoms to fully one-half of the whole mortality.

We may now proceed to enumerate the particular symptoms of the disease; but, in so doing, we shall only notice those circumstances which appear to us to present either some features of novelty, or which are not universally known or acknowledged.

*Cough.*—M. Louis observes, that some patients were only affected with this symptom in the last days of their existence, although tuberculous excavations had existed in the lungs for some time. Others (a very small number, however,) coughed but little, or after a certain time, lost the cough entirely, and which only returned a few days previous to their death. In general, the cough was aggravated at night. The force and frequency of this symptom were generally in proportion to the rapidity of the progress of the disease.

*Expectoration.*—The passage from the first to the second stage of phthisis, is marked by a remarkable change in the appearance of the expectoration: from being white, mucous, and full of air, it became opaque, greenish, deprived of air, and striped with yellow lines, which gave them a feathered appearance. Sometimes a white matter, like baked rice, was observed in the matter expectorated; but this was not a frequent occurrence. After the lapse of a greater or less period of time, these portions of white matter, as well as the striated appearance of the expectoration, ceased. It then became homogeneous, of a round form, and (as it were) torn or ragged in its circumference. It was heavy, but nevertheless did not always fall to the bottom of water, and sometimes floated on the surface of a clear fluid expectorated at the same time. Instead of a colour inclining to green, it became greyish, and of a dirty aspect. This happened generally from ten to twenty days before death; and then the matter lost its consistence and form, and was occasionally mixed with blood, and surrounded with a circle of a rose-colour. It is to be remarked, that it is only from the *union* of all these appearances that a diagnostic can be formed, since in chronic catarrh, or even in acute pulmonary catarrh, some of them may be met with: for example, the expectoration in these cases may be green, homogeneous, or opaque, but it will not be striated, nor mixed with white portions, nor round, as in phthisical subjects.

Our author makes some further observations on this subject, but they are for the most part minute, and we have not space to enter into the consideration of them.

*Hæmoptysis*, either slight or severe, existed in two-thirds of the cases, and it sometimes preceded the cough and expectoration for a greater or less space of time; and our author demands whether it is to be considered as the *avant courier* of tubercles, or a symptom denoting their presence? and he concludes by inclining to the belief that it may be looked upon in that light in general. Hæmoptysis was met with in the females, compared to the male, in the proportion of three to two. With regard to the age, this had no influence in the case of the male patients; but, in the female, one-third of those from nineteen to forty years had no spitting of blood, whilst of those from forty to sixty-nine only one-seventh part of the patients had escaped it. Neither had the age or constitution any apparent influence on the force of the hæmoptoe. In some cases, this symptom took place only once in the course of the disease, and it was very seldom repeated three or four times. In some rare cases, it appeared to have been caused by a severe accession of cough; but, generally speaking, it came on without any appreciable cause, was rarely accompanied by heat, or fever, or pain in the chest.

*Dyspnœa.*—Concerning this symptom, M. Louis offers no remarks which need detain us, and therefore we proceed to the next symptom, *Fever.*

This our author remarked always to commence from the very outset of the disease, whether acute or latent; and it appeared that its principal, if not its only cause, was the diseased condition of the lungs. Although cold chills were the most ordinary accompanying signs of fever, they were not constantly met with, and were wanting in a sixth of the subjects: these merely complained of great susceptibility to cold, and continued to be exempt from shiverings even during their residence in the hospital. In the majority of cases, these chills recurred solely in the evening, though now and then they took place at uncertain times during the day; but in no case were the two distinct shiverings, described by authors as taking place at fixed periods in the day, to be met with. These shiverings were generally followed by heat and sweat, but sometimes sweating was not observed; at others, sweating took place without any previous chills. M. Louis combats at some length the opinion of some authors, that the sweats and the diarrhœa have any correspondence with each other; but we neither perceive the utility of the discussion, and moreover we think that the fact is indisputable, however authors may contest the explanation.

Concerning the degree of *thirst* and the state of the *appetite*, there is nothing particular to remark.

The *diarrhœa* of the last days of life.—In this division our author places that which occurs from the twentieth to the fifth day preceding the death of the patient. This symptom took place in the fourth-part of the cases; in a few, it was preceded by chills and heat, or colics; but, in the majority, none of these symptoms were observable. There was always an exact correspondence between the symptoms and the lesions to which they could be attributed; for, if the diarrhœa only existed a few days before death, the ulcerations and the softening of the mucous membrane of the colon did not appear of a more ancient date. The diarrhœa was less considerable in those subjects where there was ulceration without softening of the mucous membrane, than in the cases where that existed.

The *long-continued diarrhœa* presented itself under two principal forms, either continued or remittent: the duration of this latter varied from fifteen months to forty-eight days. The remissions were of a greater or less length,—eight, ten, fifteen, or twenty days; the stools generally not numerous, and without any colicy pains. Ten cases of this description presented ulcerations in the small intestines; six, in the colon; and, with only one exception, in each instance they were small in size. The mucous membrane of the colon was very much softened in ten subjects, red and thickened in three of the cases; so that this kind of diarrhœa presented the same lesions as the preceding one. The long and continued diarrhœa lasted from one to twelve months, and sometimes more. In forty-one cases of this kind, thirty-five had ulcerations of the small intestines, and thirty-one in the large. In twelve instances, these ulcers extended throughout the whole of the small intestines. Nineteen cases of the same large ulcerations of the large intestine were found, and thirty of softening of its mucous membrane; so that, in general, where the diarrhœa was of long continuance, the intestinal ulcerations were vast and numerous: that is to say, the lesions were the same, but much more marked and ancient, than in those cases where the diarrhœa had been of long standing, but not continual.

*Emaciation* commenced, in one-half of the patients, with the first symptoms of the disease, whether it afterwards proceeded slowly or rapidly; with a third of the patients it only commenced with the fever. When diarrhœa became established, emaciation made rapid progress, and, unless some accident hastened death, it proceeded to the last degree of extenuation. Emaciation may afford a useful hint to the physician in his diagnosis in cases of latent phthisis. Where there are no local symptoms, but the patient is tormented with continued fever, with oppression and loss of flesh, this condition is generally the result of pulmonary disease. The emaciation affected equally all the tissues; the adipose cellular membrane disappeared almost entirely, and even the skin itself became thinner, and the diminution of the bulk of the muscles was not less marked.

The *countenance* had no particular expression: sometimes the face became gradually more pale, in others the colour was augmented, which appeared only rarely, and owing to particular circumstances. The surface of the body generally partook of the paleness of the face. There was occasionally a slight œdema about the ankles, and still more seldom of the whole lower extremity. The same appearance was seen now and then in the hand and arm, which announced a serous extravasation in the cavity of the chest; but these symptoms are not peculiar to phthisis.

A short chapter on the *Diagnosis of Phthisis*, including two or three cases, with the dissections, follows next in order: but it must be evident that the first period of the malady is the only one in which it is desirable to establish some precise diagnostic marks; but, on reading attentively what our author has urged upon these points, we feel compelled to say that he has added little or nothing to what has been observed before. The kind of cough, the nature of the expectoration, the dyspnœa, the wandering pains in the chest, and the imperfect sound produced by auscultation, form altogether good grounds for suspecting the existence of the malady; and that, we believe, is all that can be said, though it has been repeated again and again.

Passing by the third chapter, which occupies three or four pages only, relative to the *peripneumony* or *pleurisy* attending the few last days of the patient's existence, we come to consider the *symptoms attending ulcerations of the Epiglottis, Larynx, and Trachea.*

Of eighteen individuals affected with the first of these ulcerations, six had no lesion either of the larynx or trachea. The symptoms attending ulcers of the epiglottis were a fixed pain in the upper part of, or above, the thyroid cartilage; a difficulty of swallowing, and the rejection of fluids through the nose; the tonsils and pharynx appearing to be perfectly sound. When the larynx only is affected, neither of the two latter symptoms are to be met with. In the absence of those symptoms proper to ulcerations of the larynx, a fixed pain at the superior part of the thyroid cartilage would, perhaps, sufficiently indicate the same condition of the epiglottis; at least, the following case renders such an opinion probable.

A tailor, forty years of age, of a weakly constitution, born of parents who died at an advanced age, was admitted into La Charité on the 18th October, 1824. He had never had any serious illness; was not subject to catarrh, but had been ill about fifteen months, and had been affected with cough the whole of that period. The cough had been dry for the first two months, afterwards accompanied with abundant expectoration, and soon followed by difficulty of breathing. Three months after the first attack, violent pains had been felt in the sides of the chest, had lasted fifteen days, and had been subsequently renewed at two different times, but for a shorter period. For the two last weeks there had been slight pains in the throat, hoarseness, and difficulty in swallowing. Perspirations had only appeared by intervals, and he had not experienced any cold chills. The appetite had gradually diminished. For six months the diarrhœa had been seldom interrupted; sometimes attended with gripings, which were stronger for the first eight weeks than afterwards. The emaciation and weakness dated from the commencement of the expectoration. The patient had ceased to work for six months, and had kept his bed for two.

On the 19th of October, his condition was as follows:—Countenance pale and thin; pricking pains at the upper part of the thyroid cartilage, with a sense of dryness of that part; swallowing somewhat impeded, although the pharynx and tonsils were in a natural condition. No marked sensation in the course of the trachea. Cough moderately frequent; expectoration scanty, not entirely opaque. Chest not sonorous under either clavicle, but particularly the right. The respiration tracheal; pulse accelerated; and great sensibility to cold. Appetite almost gone. Six stools within the last twenty-four hours. Not much thirst.

These symptoms went on increasing, without any alteration worth recording, until the 1st of November, when death took place.

In giving an account of the examination of the body, we shall restrict our extracts to those appearances which bear particularly on the symptoms connected with the air-passages.

In the neck, there was œdema of the glottis, one line and a half in thickness; in the neighbourhood of the aretenyoid cartilages, much less than that in the other parts. The mucous membrane of the epiglottis was more or less red; had some ulcerations on its lingual surface, as well as the lower surface, which was of an unusual brilliant appearance, and of the same colour. The larynx was in its natural state; but the mucous membrane of the trachea was red at its lower part.

The details of two other interesting cases of the same kind occur, accompanied by the dissection of the subjects, and which offer some varieties in the appearance of the epiglottis and the neighbouring parts, such as small ulcerations at the base of the tongue or about the pharynx. In one case, the epiglottis, the lateral ligaments, and the vocal cords, were entirely destroyed; but in both the aretenyoid cartilages were perfectly sound.

The symptoms produced by *ulcerations of the larynx* varied according to the seat, the extent, and depth, of the ulcerations. Out of five individuals where these ulcerations were only found at the union of the vocal cords, one only had the voice altered from the sixtieth to the twentieth day preceding death; after which the loss of voice was complete, and there was some pain about the larynx. The other four only felt a slight degree of dryness and heat in the throat during the last weeks of their existence. In nine cases, where the ulcers were small, superficial, situated within the ventricles, between the aretenyoid cartilages, or upon the lower vocal cords, there was hoarseness, an alteration to a greater or less extent of the voice, pricking pains in the larynx, and afterwards the voice became extinct, or nearly so. These symptoms were but slightly marked, and, excepting the hoarseness, were entirely wanting in two individuals. In three cases this hoarseness commenced eight days, and in the others six or eight months, before death. The pain had also nearly the same duration. Aphonia only existed in two cases. Where the ulcers were deep, and the vocal cords more or less completely destroyed, the same symptoms occurred, only with a greater degree of intensity; but they presented the greatest difference with respect to force and duration, so that we may regard a trifling pain of some duration in the larynx, joined with some degree of alteration of the voice, as the symptoms of a superficial ulceration; whilst a violent continued pain, often very severe, followed by aphonia during one or more months, indicate deep ulcerations.

Symptoms of *ulceration of the trachea*.—However numerous these were found to be, they never gave rise to any particular symptoms. In one case only, where the mucous membrane of the trachea was destroyed throughout the whole of its fleshy portion, the patient complained, a long time before death, of a feeling of obstruction placed above and behind the sternum, and soon afterwards a sensation of heat was also felt. There was nothing in the expectoration that denoted this particular lesion. These remarks are illustrated by an interesting case; and in another, where the disease of the trachea was still more considerable, no peculiar symptom was observable. It must also be recollected, that inflammation of the mucous membrane of the trachea, without ulcerations, sometimes takes place in the progress of phthisis; but there does not appear to be any thing peculiar in the symptoms.

We now come to the fifth chapter, containing the symptoms produced by the different lesions of the *mucous membrane of the Stomach*; and the first which M. Louis mentions are those produced by the softening and thinning of this membrane. At an uncertain epoch, commonly two, four, or more months before death, the patients affected with this lesion lose their appetites, and then experience severe pains in the epigastrium. Some time after this, nausea, and then vomiting, comes on; or occasionally these symptoms took the lead, and pain followed them. These symptoms existed in different degrees in almost every case. Three cases only presented no gastric symptom, notwithstanding the depth and extent of the disease of the mucous membrane. The pain was sometimes so violent as to absorb the whole of the patient's attention: it was generally a continued pain, though there were exceptions to this. The least pressure on the epigastrium was almost insupportable; and liquids taken at a common temperature, appeared as if iced. Opium did not sensibly decrease this pain, but Seltzer water often diminished it. Notwithstanding this disorder, some patients digested light food with tolerable ease; in others, food could only be taken at some particular hour, usually in the morning. When these have persisted for a certain time, the softening and thinning of the mucous membrane of the stomach may be considered as certain.

In eight individuals, *inflammation* of the mucous membrane of the stomach was met with: this was confined to its anterior face. But the symptoms accompanying this lesion do not appear to be very distinctly marked: and, in truth, we must be permitted to say that, in these, and some other minute distinctions which our author has drawn with reference to the precise seat of some of these affections, he seems to have carried his refinements to a degree of minuteness which we are inclined to look upon with some suspicion; at all events, we do not perceive their utility in a practical point of view.

Some of the changes which our author describes are extremely rare; such, for example, as ulceration of the mucous membrane of the stomach, of which only three instances were met with; and here pain in the epigastric region, loss of appetite, and slow and imperfect digestion, are the only symptoms indicating this lesion,—symptoms common to many other appearances.

*State of the Tongue.*—The chief diseased appearances of the tongue have reference, of course, to the condition of the mucous membrane of the stomach; but in some cases the tongue was the seat of an albuminous exudation, more important to study than its mere redness. This exudation developed itself in the last period of the malady, four, eight, or ten, and sometimes even sixty, days before death; sometimes under the form of patches of two or three lines in extent, and which, uniting together, occasionally covered the tongue throughout its whole extent. Now and then it appeared in the form of little grains, separated by spaces of greater or less extent, where the tissue of the tongue was bare, easily removeable: this exudation commonly was renewed several times prior to death. In many instances, it appeared at the same time upon the tongue and on different parts of the mouth, the lips, cheeks, gums, and even the palatine arch. The tongue was almost always the seat of painful pricking sensations, and was more or less red and burning; though there were exceptions to this.

In the state of the *male and female genital organs*, we find little to notice; in the former, absolutely nothing worth extracting. With regard to menstruation, it generally ceased at a period of the disease more or less advanced. Once only it continued until death, but in a scanty and irregular manner. Our author, in mentioning the belief commonly entertained that pregnancy retards the progress of consumption, is inclined to doubt the fact; for he observes, that, during pregnancy, some of the symptoms may be more obscure, though the disease is still pursuing its accustomed course: and, again, it may be easily conceived that, after delivery, these symptoms may appear more strongly marked than during gestation; and, in fact, M. Louis mentions two cases, in which the phthisis and the pregnancy each continued their regular course. In one, the patient died twenty days after having given birth to a very robust infant.

*Cerebral symptoms.*—Nearly all the patients preserved their intellects entire until death: a few, however, in whose bodies a partial and pulpy softening of the brain was found, together with traces of inflammation of the arachnoid membrane lining the lateral ventricles, or of the tissue beneath, presented very remarkable cerebral symptoms in the last days of their existence. These symptoms were wanting in three out of six subjects affected with pulpy softening. They were only found in one case of arachnitis, and this case is detailed at length. The symptoms with which we have to do were the following, and they came on three days before death. On the first of these days, there was an almost continual sleepiness, a slight disturbance of the intellectual faculties; but no pain was complained of. In the night of the following day, there were involuntary stools ; the patient did not reply to questions, tried to rise, saying that he was about to return to his home, and fell to the ground. The next morning, at seven o'clock, his countenance appeared stupid ; the eyes were fixed, the pupils contracted ; the masseter muscles, and those of the right arm, were agitated with almost continual spasms ; the left thigh and arm were rigid, and the slight motion communicated to them produced contortions of the countenance. The loss of sense was not perfect, for the patient made efforts to show his tongue when asked to do so. The pulse was 114 in the minute, and the respiration was not at all sensibly changed. These symptoms continued, with little variation, for twenty-four hours, when the patient expired.

In the sixth chapter, M. Louis discusses the *varieties of Pulmonary Consumption,* of which he notices two, the latent and acute phthisis.

Our author commences his remarks upon *latent phthisis* with the relation of a case, from which we collect that the disease was divisable into two distinct epochs. In the first there was fever without cough : this lasted about a year. In the second period, in addition to the fever, there was cough and expectoration. M. Louis here asks, whether phthisis may be said to have existed from the first period, or whether it began with the second ? In considering that dissection showed no other organic mischief than that met with in the lungs, he is inclined to answer the question in the affirmative, especially since the fever preserved the same character in both periods, and as there was no previous catarrh. He is likewise disposed to think that tubercles can become developed in the lungs without any such exciting cause. Another curious fact connected with this case is the loss of appetite for upwards of three years, without any corresponding alteration being discovered in the mucous membrane of the stomach.

Five analogous cases next present themselves, in which the same minuteness of description, and the same accuracy of anatomical investigation, occur ; but they add but little to the principal facts mentioned above, and are of a length that defy compression.

With respect to the proportion in which these latent cases of consumption are met with, we are told that, of 123 cases of phthisis, eight, or the fifteenth part, were specimens of this species. This proportion, though considerable in itself, is much less so than that which really exists, if, as we have observed before, the hœmoptoe which precedes the cough and expectoration is the effect, and not the *avant courier*, of the tubercles. This kind of hœmoptoe had, in fact, preceded the other symptoms in seven cases where the progress of the complaint had been considered as regular.

The eight cases of latent consumption above mentioned naturally divide themselves into two orders: in the one, the tubercles had existed a greater or less space of time before they caused either cough or expectoration, or even any general symptoms of consequence;. in the other, they gave place to general symptoms of great intensity, such as fever, loss of appetite, and emaciation, long before there existed either cough or expectoration. In the one case, the weakness of the symptoms prevented the state of the lungs being exactly known or suspected; but, in the other, both on account of the difficulty of assigning these symptoms to any other lesion, as well as owing to the frequency of consumption, the existence of tubercles might be suspected; which suspicion an attentive search into local symptoms, or especially auscultation and percussion, would confirm.

*Acute phthisis.*—In the first case of this kind recorded by M. Louis, the disease ran its course in thirty-five days; the cough only existed twenty-five. The patient was a girl of eighteen, had not been accustomed to take cold, and had been only fifteen days ill when admitted into La Charité. In this case it is to be remarked, that mediate auscultation entirely failed in detecting any pulmonary disease. Three other cases of acute phthisis are next recorded, and in none of these instances was death delayed beyond the fiftieth day. There was no hæmoptysis in either case, and difficulty of respiration preceded, in one or two instances, the coming-on of the croup and expectoration: nevertheless our author, with the caution and candour which marks the whole work, observes, that the number of his observations has been too limited to enable him to give a general description of this form of consumption, or to describe the diagnostic signs of its first period; although he thinks it may be dreaded in those cases where patients are seized suddenly with dyspnœa without any apparent cause; if these symptoms go on increasing notwithstanding the application of appropriate remedies; and there are no symptoms of other pulmonary affections, such as peripneumony, pleurisy with effusion, or suffocating catarrh. In these cases, mediate auscultation contributes somewhat to the diagnosis. In the second period, no doubt as to the existence of the malady can exist; since the changed appearance of the expectoration, and the state of the respiration, sufficiently explains the nature of the attack. Notwithstanding the rapidity of its progress, this disease, nevertheless, gives rise to those secondary disorders observed when its progress is more slow; such, for example, as ulcerations of the mucous membrane of the epiglottis and trachea, of that of the œsophagus and small intestines, stomach, &c.

Chapter 7th. *Symptoms of the perforation of the lungs by a tubercle opening into the cavity of the pleura.*—This perforation, pointed out by M. Laennec, is met with under two principal forms. Sometimes the tuberculous excavation, open in one or other of the pleuræ, communicates with the bronchiæ; at other times, this communication does not take place. In either case, the precise moment when the perforation happens is marked by severe symptoms, quite sufficient to establish a diagnosis. These remarks are illustrated by the relation of seven cases of this description, in which the sudden accession of pain was among the most prominent symptoms. This pain varies in its situation; in one subject it was referred to the spinal column: it differs also in degree of intensity, and is commonly attended with a difficulty of respiration, and an inexpressible anxiety, followed by all the marks of acute pleurisy. The detail of one case proves that a sensation of anxiety and suffocation, coming on in a sudden manner, may, independently of pain, lead to the suspicion of the same occurrence having taken place; but here again the sensations must be suddenly felt.

It is remarkable that the interval that has elapsed between these perforations and death, have been very various, differing from sixteen hours to thirty-six days, and it is not easy to explain why this should be. It does not appear to have been influenced by treatment, nor by the extent of the excavation, and consequent extent of the extravasation. The perforation took place at the same spot in five of the eight cases,—that is, opposite to the angle of the third or fourth rib. It is no less remarkable that seven out of eight instances took place on the left side, where, in fact, the tubercular affection is rather more frequent and more advanced than on the right side.

The subject of *Sudden Deaths* occupies the eighth chapter.—Our author observes, that the preceding facts show how many circumstances, foreign to the tubercular affection of the lungs, accelerate the death of consumptive patients; but there are cases in which it takes place still more suddenly. Sometimes the cause is made apparent by an examination of the body; at others, the most scrupulous researches lead to no satisfactory conclusion. In the first case of this kind, death appeared to be produced by the rapidity with which the lungs had become hepatised, and consequently unfit for respiration. In fact, thirty-six hours before the death of this patient, the left side of the chest sounded properly throughout its whole extent, so that the whole, or nearly so, of the lung of that side must have passed into the second degree of inflammation within that space of time, according to our author; but then we must recollect that this opinion is founded upon the infallibility of mediate auscultation. In a second case where death took place suddenly, no diseased appearances, capable of accounting for it, were discoverable either in the chest or abdomen; but there was considerable œdema of the glottis, rather more considerable on the right than on the left side, and to this M. Louis is inclined to attribute the sudden dissolution of the patient. In answer to those who doubt this explanation, he replies by relating the case of a young man attacked with severe fever, and who died in the midst of a frightful suffocation, accompanied with a hissing inspiration, which only appeared two hours before his death. At the opening of the body, the glottis was found œdematous, and to the same extent as in the case of phthisis above mentioned. Our author has observed but two other cases of œdema of the glottis in consumptive patients, which, considering the frequency of ulcerations of the epiglottis and larynx, he thinks is singular.

In the second division of this chapter, we have four cases detailed, in none of which could the sudden death of the patient be accounted for by the most minute anatomical investigation of the body; though in two of the subjects considerable softening of the brain was observed, yet not enough of itself to account for the suddenness of their death.

Chapter the ninth treats of *the Causes of Phthisis;* under which head M. Louis examines the influence of sex, of peripneumony, of pleurisy, and of pulmonary catarrh, upon the production of consumption.

First, with regard to the influence of *sex.*—Of the 123 cases observed by M. Louis in the space of three years, 66 were females and 57 males; so that, at the first view, it appears that the former are more subject to consumption than the latter: this appears to be strengthened by another fact, which is this—in an equal number of men and women who died of chronic affections, and not of phthisis, a certain number of tubercles were found in the lungs, 25 times in the female, and only 15 in the male; so that, uniting both these facts, the proportion appears to be between male and female, as 70 to 92.

Influence of *peripneumony and pleurisy.*—Of 80 individuals, whose diseases, prior to the attack of phthisis, were carefully inquired into, three had experienced the year preceding an attack of peripneumony, and four had had the same disease some years before the appearance of the phthisical symptoms, without having been more subject to colds in consequence, and without suffering any alteration in the state of their respiration: all of them were of a lymphatic temperament and feeble constitution; so that, taking all circumstances into consideration, it would not appear that either of the above diseases had any marked influence in producing consumption. M. Louis is well aware that this conclusion appears to be at variance with a crowd of facts, and especially with the observations of M. BROUSSAIS: but, says our author, pleurisy and peripneumony, either acute or chronic, are very common diseases among soldiers; and M. Broussais, having opened the bodies of a great number of subjects who died of one or other of these diseases, finding tubercles in the lungs of many of them, has concluded that they were the cause both of the pleurisy and peripneumony; but it is easy to perceive that, without a just comparison being made, and regular tables of mortality being drawn up, containing the number of those in whom tubercles in the lungs have been found after death, and who have died at the same age, in civil as well as in military hospitals, this question cannot be fairly answered. There are many other reasons also given by our author, which afford presumptive evidence of the justness of his conclusions.

Neither, in his opinion, is the influence of *pulmonary catarrh* more clearly made out than that of the above mentioned complaints. Out of 80 individuals, who gave a distinct account of their maladies prior to the commencement of phthisis, only 23 were subject to pulmonary catarrh. This view of the matter is strengthened by the consideration that females, who are more subject to consumption than males, are less frequently attacked by pulmonary catarrh; so that the sex which seems the most exposed to phthisis is the least subject to one or other of these inflammatory affections, in the proportion of one to three.

The influence of *clothing*, particularly of wearing stays, is, according to our author, an assertion without proof; but we are surprised that he did not include in this article some remarks upon the custom of the female world to lessen the quantity of clothing when the temperature is actually lowest,—we mean at night; and the contrast between the heated ball-room and the external air. To these causes combined we may certainly attribute many fatal cases in this country, and especially in the upper ranks of life.

Regarding the influence of *hereditary predisposition*, our author observes, that a tenth part of the subjects were born of parents who, on one side or other, had apparently died of consumption; and he is inclined to believe that this proportion is even below the truth.

The influence of *age* is sufficiently marked. The number of those who die of consumption between twenty and forty years of age, is much more considerable than from forty to sixty; and M. Louis's remarks go completely to confirm those made by BAYLE on this point.

A few pages (as we before stated) on the *treatment* of phthisis conclude the volume: they certainly need not detain us, for they evince no superiority of practice, to which, indeed, M. Louis lays no claim.

## Figures and Tables

**Fig. 1. Fig. 2. f1:**